# Papillary Thyroid Cancer in a Struma Ovarii in a 17-Year-Old Nulliparous Patient: A Case Report

**DOI:** 10.3390/diagnostics10010045

**Published:** 2020-01-15

**Authors:** Agnieszka Gonet, Rafał Ślusarczyk, Danuta Gąsior-Perczak, Artur Kowalik, Janusz Kopczyński, Aldona Kowalska

**Affiliations:** 1 Collegium Medicum, Jan Kochanowski University, IX Wieków Kielc Av.19, 25-319 Kielce, Poland; agonet@poczta.onet.pl (A.G.); kazzerr@gmail.com (R.Ś.); aldonako@onkol.kielce.pl (A.K.); 2 Endocrinology Clinic, Holycross Cancer Center, S. Artwińskiego St. 3, 25-734 Kielce, Poland; 3 Molecular Diagnostics, Holycross Cancer Center S. Artwińskiego St. 3, 25-734 Kielce, Poland; artur_kowalik@yahoo.com; 4 Surgical Pathology, Holycross Cancer Center, S. Artwińskiego St. 3, 25-734 Kielce, Poland; janusz_kopczynski@yahoo.com

**Keywords:** malignant struma ovarii, papillary thyroid cancer, teratoma, thyroidectomy, immunohistochemistry

## Abstract

Introduction: Struma ovarii accounts for 2% of mature teratomas. Struma ovarii is diagnosed when thyroid tissue accounts for >50% of the teratoma. Malignant transformation is rare, occurring in <5% of struma ovarii cases. Case presentation: A 17-year-old patient was diagnosed with papillary thyroid cancer in struma ovarii. The patient exhibited menstrual disorders. Abdominal and pelvic CT revealed a 17 cm mass in the left adnexa. Laparoscopic removal of the left adnexa with enucleation of right ovarian cysts was performed. Histopathological diagnosis was a follicular variant papillary carcinoma measuring 23 mm in diameter. Immunohistochemical positive expression of CK19, TTF-1, and thyroglobulin (Tg) confirmed the diagnosis. Molecular analysis detected the *BRAF K601E* mutation in ovarian tumor tissues. Preoperative serum Tg concentration was >300 ng/mL, which decreased to 38.2 ng/mL after gynecological surgery with undetectable anti-Tg antibodies. The patient underwent total thyroidectomy with no cancer detected on histopathological examination. The patient was treated with I-131 and showed no recurrence 4 years after the diagnosis. Conclusions: Malignant struma ovarii is diagnosed by surgery. Because papillary carcinoma in struma ovarii is rare and there are no guidelines regarding the management of this type of cancer, therapeutic decisions should be made individually based on clinical and pathological data.

## 1. Introduction

Germ cell tumors account for 15–20% of ovarian tumors, and most are mature teratomas [1]. Struma ovarii is characterized by the presence of >50% of thyroid tissue and accounts for 2–5% of cases of mature ovarian teratomas and 1% of all ovarian tumors [2,3,4,5]. Struma ovarii can occur in women of all ages, but it is most common in women in the fourth to sixth decades of life [3,6,7]. Although tumors are usually benign, <5% of all struma ovarii undergo neoplastic transformation, and the underlying mechanism remains unclear [8,9,10]. Similar to cancers of the thyroid gland, the most malignant type of struma ovarii is well differentiated thyroid cancer, and the most common is the papillary type (70%), whereas the follicular type (30%) is less common [2,7]. The clinical presentation of struma ovarii is nonspecific and similar to the clinical picture of other ovarian tumors. The most common symptoms are the presence of a pelvic tumor, lower abdominal pain, abnormal menstrual cycles, vaginal bleeding, ascites, and deep vein thrombosis [11,12,13]. Symptoms of thyroid dysfunction such as hyperthyroidism occur in 5–8% of cases [2,6,9]. Malignant struma ovarii metastasis occurs in 5–23% of cases and spreads via lymphatic and blood pathways. It mainly occurs in the abdominal cavity, although blood-borne metastasis can occur in the liver, lungs, brain, bones, and the opposite ovary [10,14,15]. The tumor is often accidentally diagnosed during an abdominal/pelvic ultrasound/computed tomography (CT) scan or during surgery for other reasons [11,12,13]. Because of the rarity of the disease, there is little data on the criteria for optimal diagnosis, treatment, and observation. Surgical treatment of malignant struma ovarii includes abdominal hysterectomy and bilateral salpingo-oophorectomy with omentectomy. Unilateral salpingo-oophorectomy or cystectomy is recommended for women who wish to retain fertility [11,15]. For metastatic struma ovarii, the consensus is aggressive treatment including total thyroidectomy and adjuvant treatment with radioactive iodine (I-131); however, the role of thyroidectomy and adjuvant I-131 treatment in non-metastatic struma ovarii remains controversial [9,15,16,17,18]. Here, we present the case of a 17-year-old girl with a follicular variant of papillary thyroid cancer arising in struma ovarii who was diagnosed after surgery to remove the left adnexa due to a large tumor in the left ovary.

## 2. Case Presentation

**Figure 1:**
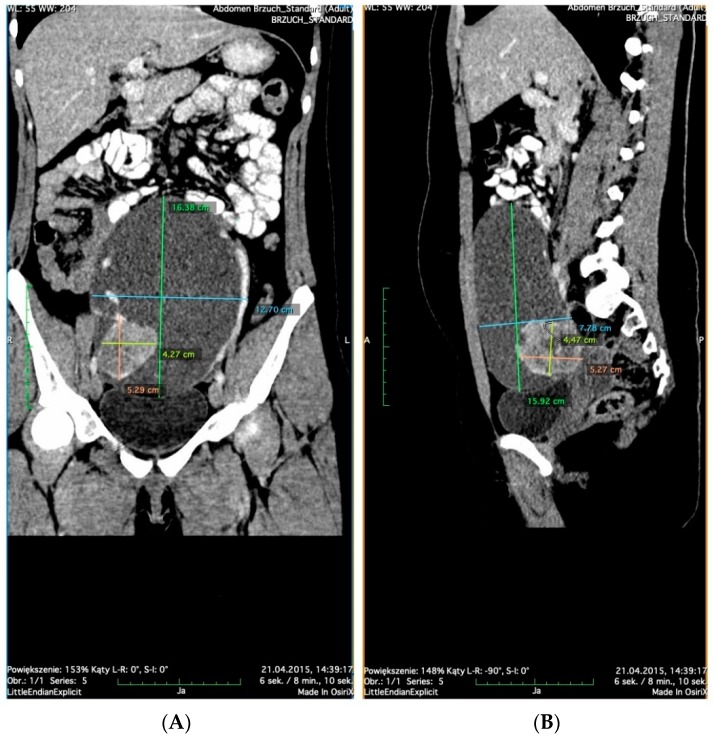
CT scans of the abdominal cavity and pelvis, which revealed a vast cystic-solid lesion: (**A**) coronal CT; (**B**) sagittal CT.

A 17-year-old girl, nullipara, virgin, came to the Outpatient Gynecological Clinic with complaints of menstrual disorders with polymenorrhea. The gynecological interview revealed that the first menstrual period at 12 years old was spontaneous, with regular menstrual periods every 28 days. For the past 2 years, menses had been scanty, occurring every 2 weeks. Physical examination showed the presence of a large mass in the lower abdomen reaching the navel. Ultrasound (US) examination through the abdominal wall revealed a cystic-solid tumor measuring approximately 15 cm adjacent to the left ovary. To clarify the diagnosis, a multi-phase CT scan of the abdominal cavity and pelvis with contrast was performed, and a blood sample was taken for analysis of basic tumor markers. CT scan of the abdominal cavity and pelvis confirmed a vast cystic-solid lesion measuring 8 × 13 × 17 cm, including a solid fragment measuring 4 × 5 × 5 cm, originating from the left adnexa, suggesting the presence of a tumor in the left ovary (Figure 1). We confirm that the written informed consent was obtained from the participant.

**Figure 2:**
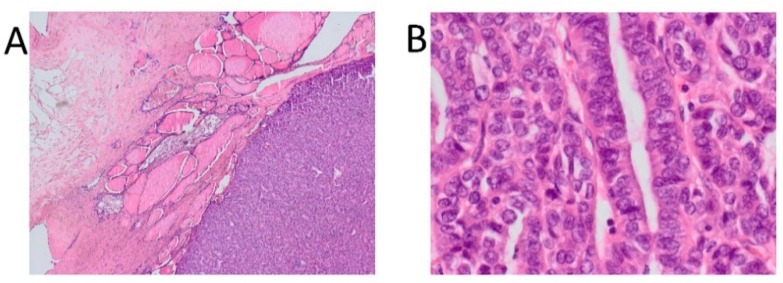
Gross photographs of surgical specimens. (**A**) 40× magnification, hematoxylin and eosin (H&E) staining; the ovarian capsule is visible on the top-left side, struma ovarii is visible in the middle of the image, and violet-stained follicular variant of papillary carcinoma on the right. (**B**) 200× magnification, H&E staining, papillary carcinoma; visible cytological features of the cancer include cell overlapping and cell nuclei clearing.

Basal tumor markers were as follows: CA-125 17.3 U/mL (range 0.0–35); beta HCG <1.2 mlU/mL (range 0.0–5.0); CA19-9 3.54 U/mL (range 0.0–37); CEA <0.5 ng/mL (range 0.0–5.0). The patient underwent laparotomy to remove the left adnexa because of the large tumor mass, and a section was taken from the omentum. During the procedure, approximately 5 cm of the right ovarian cyst was detected and enucleated. The collected material was examined intraoperatively, which showed no presence of cancer. The results of histopathological examination revealed struma ovarii malignum “post intram”. A follicular variant of papillary carcinoma (23 mm in size) was detected within the left struma ovarii. No infiltration of the capsule was found (Figure 2).

**Figure 3:**
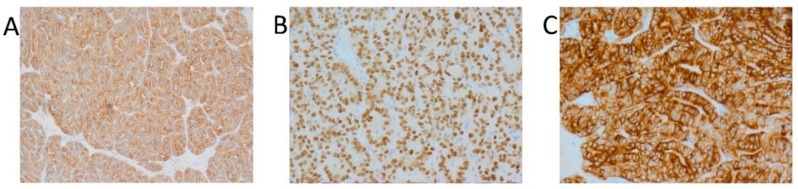
Immunohistochemical (IHC) staining of PTC in struma ovarii (**A**) 100× magnification; immunoreactivity for thyroglobulin, (**B**) 200× magnification; immunoreactivity for TTF-1, (**C**) 200× magnification; immunoreactivity for CK19.

No tumor cells were detected in the enucleated right ovarian cyst or in a slice of the sample from the omentum. The diagnosis of cancer was confirmed by immunohistochemistry. Immunohistochemically, the tumor cells were strongly positive for thyroglobulin (Tg), transcription thyroid factor type 1 (TTF-1), and cytokeratin (CK19), and were negative for estrogen receptor (Figure 3).

**Figure 4:**
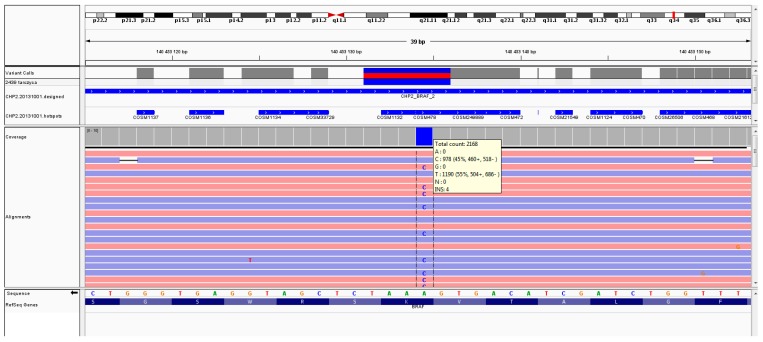
Screen shot of the next-generation sequencing missense mutation p.K601E (c.1801A>G p. Lys 601Glu) detected in *BRAF*. NGS data showing reads (- strand designated in blue, + strand in red) mapped to the oncogene reference sequence are shown below the panel box depicted by Integrative Genomics Viewer. Because of the opposite DNA strand orientation, the mutation is presented as T>C instead of A>G on the figure.

Molecular analysis of DNA obtained from the resected tumor tissues was performed using next generation sequencing (NGS). The hot spots in 50 genes in which mutations are present in various types of cancer were sequenced (NGS, sequencing in Ion Torrent technology) using the Cancer Panel v2* kit (Thermo Fisher Scientific). The panel includes the following genes: *ABL1*, *EZH2*, *JAK3*, *PTEN*, *ACT1*, *FBXW7*, *IDH2*, *PTPN11*, *ALK*, *FGFR1*, *KDR*, *RB1*, *APC*, *FGFR2*, *RET*, *ATM*, *FGFR3*, *KRAS*, *SMAD4*, *BRAF*, *FLT3*, *MET*, *SMARCB1*, *CDH1, GNA11*, *MLH1*, *KIT*, *SMO*, *CDKN2A*, *GNAS*, *MPL*, *SRC*, *CSF1R*, *GNAQ*, *NOTCH1*, *STK11*, *CTNNB1*, *HNF1A*, *NPM1*, *TP53*, *HRAS*, *NRAS*, *VHL*, *ERBB2*, *IDH1*, *PDGFR*, *ERBB4*, *JAK2*, *PIK3*, and *EGFR*. NGS identified a mutation in the *BRAF* p.K601E gene (c.1801A > G p. Lys 601Glu), whereas no mutations were detected in other genes (Figure 4).

The patient was referred to the Endocrinology Clinic to determine further treatment. The patient’s physical examination showed no abnormalities. The serum Tg, TSH, aTg, and TPO levels were determined from the serum, which was frozen before gynecological surgery. Tg concentration before surgery was >300 ng/mL (range 0.0–55 ng/mL), and it decreased to 38.30 ng/mL after surgery; serum TSH was 1.92 µIU/mL (range 0.35–4.94); no serum Tg or thyroid peroxidase antibodies were detected (anti-Tg <20 IU/mL, range 0.0–40.0; anti-TPO <10 IU/mL, range 0.0–35.0). Thyroid US examination showed a hypoechogenic focus in the lower pole of the left lobe. Fine needle aspiration biopsy of the focus performed under US detected a benign lesion (category II, according to the Bethesda system, 2009) [19]. Whole-body PET/CT 18F-FDG examination was performed, which did not reveal hypermetabolic proliferative areas. Whole-body diagnostic scintigraphy with I-131 showed a normal pattern of marker uptake in the thyroid, with no pathological marker accumulation outside the thyroid. Because of the PTC in the struma ovarii measuring >2 cm [2], and relatively high metastasis and recurrence rates (5–23% and 7.5–35%, respectively) [2,9,15], the decision was made to remove the thyroid and perform I-131 adjuvant therapy. Postoperative histopathological examination showed no malignant changes in the thyroid. One month after thyroid surgery, adjuvant I-131 therapy was performed with a dose of 1,100 MBq I-131. Whole-body, post-therapeutic scintigraphy performed 72 and 96 h after I-131 administration showed an area of increased I-131 uptake in the thyroid bed projection with no other pathological uptake areas. The TSH concentration before therapy with I-131 was 72.5 µIU/mL; serum Tg concentration was 7.68 ng/mL in the absence of anti-Tg antibodies. Levothyroxine treatment was started at a dose of 1.6 µg/kg/day, and TSH and Tg levels were assessed after 3 months, increasing the dose to 1.98 µg/kg/day until the appropriate TSH value of 0.1–0.4 µIU/mL (mild suppression) was reached until follow-up diagnosis after 12 months [20]. Two weeks after I-131 treatment, the patient underwent laparoscopic appendectomy because of acute appendicitis. After diagnostics performed 12 months after the primary treatment (surgery + I-131) under conditions of stimulation with exogenous recombinant human TSH (rhTSH), the patient met the criteria of indeterminate response to treatment (stimulated Tg of 1.04 ng/mL with undetectable anti-Tg antibodies, and absence of morphological features of the disease on neck US and I-131 whole-body scintigraphy) in accordance with the response criteria of the American Thyroid Association guidelines [21]. During the 4 years of follow-up, the patient was treated with levothyroxine at a dose that maintained the concentration of TSH in the range of 0.1–0.4 µIU/mL (mild suppression) [20]. At present, the patient shows no signs of recurrence (Tg/L-T4 concentration <0.04 ng/mL with undetectable anti-Tg antibodies and the absence of morphological features of the disease on US).

The patient also remains in gynecological control. After gynecological surgery, menstruation occurred every 30–35 days. Two years after gynecological surgery, the patient showed increasing features of hyperandrogenization in the form of hirsutism of the skin, including the face, chin, abdomen, and along the linea alba, and she underwent hormonal and imaging tests of the remaining ovary. BMI was 24 kg/m^2^, and blood pressure was 100/60 mmHg. The results of hormonal tests showed no deviations (androstenedione 3.43 ng/mL (range 0.30–3.70); total testosterone 0.39 ng/mL (range 0.14–0.49); free testosterone 1.780 pg/mL (range 0.3–3.05); DHEAS 454.5 µg/dl (range 61.2–493.6); FSH 5.14 mIU/mL (range 2.8–11.3); LH 4.9 mIU/mL (range 1.1–11.6); estradiol 41 pg/mL (range 21–251); total prolactin 179 mIU/L (range 40–530); 17-OH-Progesterone 0.766 ng/mL (range 0.27–2.31); beta HCG <1.2 mIU/mL (range 0.0–5.00); Ca-125 8.2 U/mL (range 0.00–35); AMH 3.26 ng/mL (range 1.52–9.95); SHBG 37.42 nmol/L (range 25–135); HOMA 1.4 (range <2.5); features of PCO on US). An oral contraceptive tablet was included in the treatment (ethinylestradiolum 0.02 mg + desogestrelum 0.15 mg). Currently, the patient has discontinued oral contraception because of maternity plans. She remains in endocrine and gynecological control.

## 3. Discussion

Most of the struma ovarii in which thyroid-type carcinomas arise are unilateral and more often affect the left ovary [11], as was the case in the presented patient. The average age of patients with struma ovarii is approximately 43 years [7], although there are cases in the literature of struma ovarii diagnosed in a child of 10 years [22] and in younger women (22 and 25 years) [23,24], whereas the present patient was a teenager. The most common type of thyroid cancer detected in this type of teratoma is papillary thyroid cancer [2,7]. The present patient had a variant of follicular papillary thyroid cancer that ranks second in incidence after the classical variant [15,25]. Distant metastases of malignant struma ovarii are rare and occur in approximately 5% of cases [3,9]. However, they are more common in the abdominal cavity, accounting for up to 23% of cases [14]. In the present patient, no metastases were found. The criteria for malignancy in struma ovarii are disputable. Histopathological diagnosis of malignant struma ovarii is in accordance with the guidelines for the diagnosis of primary thyroid cancer based on the characteristic microscopic features after staining the sample with hematoxylin–eosin. Such features include “ground glass nuclei”, intranuclear inclusions, and vascular invasion [11,26]. Pardo-Mindan et al. noted that nuclear changes alone were not sufficient to make the diagnosis because atypical cells often occur in non-cancerous lesions [27]. However, these authors also emphasized that the diagnosis of malignancy should include features such as the presence of capsular invasion or extraovarian spread into the peritoneum [27]. These features were not present in the present patient on histological examination. The diagnosis of thyroid cancer in struma ovarii can be difficult, and immunohistochemical analyses (e.g., TTF-1, Tg, and CK19) can help in making a diagnosis [28]. In the present patient, we confirmed the positive expression of CK19, TTF-1, and Tg in the tumor, which suggested a malignant process. In addition, molecular analysis can help distinguish between benign and malignant tumors. The occurrence of molecular abnormalities within the struma ovarii has been documented in the literature. In 2004, Trovisco et al. showed that *BRAF* mutations are associated with the occurrence of some histological types of papillary thyroid cancer. These authors also reported that the *BRAF* K601E mutation is unique to the follicular papillary thyroid cancer variant [29]. Schmidt et al. showed the presence of *BRAF* mutations (V600E, K601E, and a deletion/substitution TV599-600M), suggesting the presence of a common pathogenetic pathway for all papillary thyroid cancers regardless of location [30]. Similar conclusions can be derived from the work of Goffredo et al., who demonstrated the coexistence of malignant struma ovarii with thyroid cancer in approximately 9% of cases [7]. Mutations in the *BRAF* gene were reported by other authors [31,32]. Point mutations in the *NRAS* [33,34] and *HRAS* [35] genes, as well as *RET/PTC* rearrangements [36], were also reported. We detected the *BRAF* K601E mutation in our patient’s DNA.

There is an ongoing discussion regarding the therapeutic management of malignant struma ovarii. Treatment guidelines remain to be established, and the optimal surgical treatment and postoperative management are controversial. Recommendations are based on individual case reports or review work. Surgery might consist of total hysterectomy with excision of the adnexa and ovaries or sparing surgery including unilateral oophorectomy [11]. Radical surgery is appropriate for postmenopausal women or those who are not planning to become pregnant, whereas conservative surgery is often the treatment of choice for women who have maternity plans; however, this is only applicable to unilateral disease without capsular invasion or metastases [15].

Because our patient was young and nulliparous, gynecological surgery was limited to unilateral oophorectomy to preserve fertility and hormonal function in the second ovary. The opposite ovary should be examined during surgery to exclude pathological changes. In the present patient, a tumor was detected during gynecological surgery in the opposite ovary that was ultimately identified as a simple cyst postoperatively. In cases of malignant struma ovarii with distant metastases, the consensus is that a more aggressive treatment approach (total hysterectomy with bilateral excision of the adnexa and ovaries, omentectomy, total thyroidectomy, and I-131 therapy) is warranted. The goal of I-131 treatment is ablation of thyroid remnants and destruction of metastatic foci of thyroid cancer. This enables monitoring for disease using whole-body I-131 scanning and analysis of serum Tg levels [15]. One of the most disputed issues is the use of preventive thyroidectomy with postoperative I-131 administration in patients with a non-metastatic malignant struma ovarii [15]. Because malignant struma ovarii can coexist with thyroid cancer, Janszen et al. and Tzelepis et al. recommend performing a total thyroidectomy followed by I-131 treatment to remove a possible primary thyroid cancer and micrometastases. In addition, this procedure allows the determination of Tg as a follow-up marker [37,38]. Many authors support this approach with the aim of reducing the rate of recurrence and mortality [8,9,11,39,40,41]. Jean et al. provided data supporting this strategy as the optimal treatment; these authors reported a recurrence rate of 21% among 42 patients with malignant struma ovarii who had undergone surgery alone. By contrast, DeSimone et al. reported the results of postoperative I-131 treatment and showed considerably better outcomes: Of 24 patients, 16 patients did not receive I-131 therapy after surgery, whereas eight received I-131. There were eight recurrences, which all occurred in patients who did not receive I-131 treatment. These patients were then treated with I-131, which led to a complete response in seven of the eight patients [9].

Yassa et al. emphasized the role of patient stratification according to the risk of recurrence in the management of malignant struma ovarii. In patients with a low risk of recurrence (i.e., primary tumors smaller than 2 cm limited to the ovary and without aggressive histopathological features), they propose unilateral salpingo-oophorectomy and levothyroxine treatment to maintain serum TSH levels at 0.1–0.5 mIU/L [2].

However, high-risk patients with tumors larger than 2 cm or aggressive histopathological features require additional treatment (i.e., total thyroidectomy and adjuvant I-131 therapy) to detect and potentially treat recurrences [2]. In our institute, we use this treatment approach. The management and prognosis can also be influenced by the size of the struma ovarii and the growth rate [42].

Regardless of the type of treatment, Makani et al. recommends long-term monitoring for at least 10 years including clinical monitoring and assessment of Tg levels [14]. The biological behavior of these cancers is unclear. In the largest series of patients studied (57 cases treated with various surgical procedures and adjuvant I-131, who were followed-up for 25 years), the recurrence rate was 7.5% [15]. In another study, the recurrence rates were as high as 35%, which underscores the crucial role of long-term follow-up [9].

Well differentiated thyroid cancer in struma ovarii is rare and has a high percentage of metastases and recurrences [2,9,15]. To date, no guidelines have been established for the treatment of thyroid cancer in this location. However, the evidence-based recommendations for management of thyroid cancer in the thyroid gland were developed by ATA in 2015 [21]. Due to the common origin of thyroid cancer in the thyroid gland and thyroid cancer in struma ovarii, we have decided to use the treatment method in accordance with the ATA recommendations [21,30], but it should be emphasized that each case should be considered individually and that there are no arguments to date for or against the use of ATA guidelines in the treatment of thyroid cancer in struma ovarii. Further research into this rare disease is needed to reach definitive conclusions.

## 4. Conclusions

Malignant struma ovarii is usually diagnosed during surgery. Immunohistochemistry and molecular tests can help determine risk groups. Because papillary carcinoma in struma ovarii is rare and there are currently no guidelines for the management of this type of cancer, therapeutic decisions should be made individually based on clinical and pathological data.
